# Whole‐exome sequencing and immunohistochemistry findings in von Hippel–Lindau disease

**DOI:** 10.1002/mgg3.880

**Published:** 2019-07-17

**Authors:** Xiaopeng Guo, Lu Gao, Xiafei Hong, Dan Guo, Wenyu Di, Xiaoman Wang, Zhiqin Xu, Bing Xing

**Affiliations:** ^1^ Department of Neurosurgery Peking Union Medical College Hospital, Chinese Academy of Medical Sciences and Peking Union Medical College Beijing P.R. China; ^2^ Department of General Surgery Peking Union Medical College Hospital, Chinese Academy of Medical Sciences and Peking Union Medical College Beijing P.R. China; ^3^ Peking Union Medical College Hospital, Chinese Academy of Medical Sciences Corelabs Beijing P.R. China; ^4^ Clinical Bio‐bank Peking Union Medical College Hospital, Chinese Academy of Medical Sciences and Peking Union Medical College Beijing P.R. China; ^5^ Department of Pathology Xinxiang Medical University First Affiliated Hospital Weihui P.R. China; ^6^ State Key Laboratory of Medical Molecular Biology, Institute of Basic Medical Sciences Chinese Academy of Medical Sciences and Peking Union Medical College Beijing P.R. China

**Keywords:** hemangioblastoma, phospho‐S6 ribosomal protein, *VHL* gene, von Hippel–Lindau disease, whole‐exome sequencing

## Abstract

**Background:**

von Hippel–Lindau (VHL) disease has a hereditary, autosomal dominant pattern, and multiple tumors can develop in multiple organs of a single patient. However, the exact mechanisms of tumorigenesis are unclear, and further studies are needed to clarify whether the same signaling pathways are involved in different VHL‐related tumors.

**Methods:**

Whole‐exome sequencing (WES) of tumor and paired peripheral blood samples were performed for a VHL disease pedigree. A bioinformatics analysis was conducted to identify candidate somatic single‐nucleotide variants (SNVs) present in all tumor tissues. Sanger sequencing was then used to validate the SNVs identified using WES. Immunohistochemistry was performed to analyze components of the mTOR pathway, which was abnormally activated in tumor tissues.

**Results:**

Two hemangioblastomas and two renal cell carcinomas were sequenced. The bioinformatics analysis revealed a *VHL* somatic variant in all tumors; no other SNV was detected. Immunohistochemistry showed the abnormal expression of the phospho‐S6 ribosomal protein in the hemangioblastomas, but not in the renal clear cell carcinomas.

**Conclusion:**

Except for a SNV in the *VHL* gene, no other somatic SNVs were detected using WES. The phospho‐S6 ribosomal protein in the mTOR pathway is a potential target in VHL‐related cerebellum hemangioblastomas.

## 1 INTRODUCTION

von Hippel–Lindau (VHL, OMIM accession number: 193300) disease is a rare, hereditary, autosomal dominant, neoplastic disease with a prevalence of 1/38,951‐1/53,000 (Binderup, Galanakis, Budtz‐Jorgensen, Kosteljanetz, & Bisgaard, 2017; Maher et al., 1991; Neumann & Wiestler, 1991). The disease was named after the German ophthalmologist von Hippel (von Hippel, 1904) and Swedish pathologist Lindau (Lindau, 1926). The majority of patients with VHL disease have a family history, although approximately 20% carry a sporadic de novo variant causing the disease (Richards et al., 1995). Highly vascular tumors, including central nervous system (CNS) hemangioblastomas, retinal hemangioblastomas, renal clear cell carcinoma (RCCC), and pheochromocytomas, are the most common neoplasms in patients with VHL disease. Other lesions include pancreatic neuroendocrine tumors, endolymphatic sac tumors, renal cysts and pancreatic cysts. Patients with a family history are clinically diagnosed with VHL disease based on the confirmed presence of only one of the aforementioned tumors; in patients without a family history, a diagnosis is only conclusive if two of the aforementioned neoplasms (e.g., two hemangioblastomas, one hemangioblastoma plus one RCCC or pheochromocytoma) are confirmed (Maher et al., 1991). VHL disease is present in 30% of clinical patients with CNS hemangioblastomas, half of patients with retina hemangioblastomas, and 1%–2% of patients with RCCCs (Gerlinger et al., 2014; Kaelin, 2004; Maher et al., 1991; Neumann et al., 2002; Richards et al., 1995).

Because of the rarity of the disease and the complexity and atypia of the clinical phenotypes, a definitive diagnosis and timely treatment of VHL disease are challenging based only on clinical manifestations and radiological images. However, following the identification of the VHL gene and its associated pathways, a genetic diagnosis using Sanger and multiplex ligation‐dependent probe amplification of the VHL gene has increasingly been used to confirm a clinically suspected VHL diagnosis (Dandanell, Friis‐Hansen, Sunde, Nielsen, & Hansen, 2012). The gene, which encodes a tumor suppressor, was mapped to chromosome 3 in 1988 and isolated in 1993 (Latif et al., 1993; Seizinger et al., 1988). The VHL protein (pVHL), inhibits tumor cell proliferation by inducing the ubiquitination and degradation of hypoxia‐inducible factors (HIFs) (Gossage, Eisen, & Maher, 2015; Nielsen et al., 2016). VHL exon deletions, missense substitutions, and other variants can result in the formation of a truncated protein and the accumulation of HIFs, leading to increased replication and expression of vascular endothelial growth factor and growth of highly vascular VHL‐related tumors (Gossage et al., 2015; Nielsen et al., 2016). In addition to VHL gene variants, copy number variations (CNVs) and/or loss of heterozygosity (LOH) are also essential genetic mechanisms of VHL disease (Fei et al., 2016; Tory et al., 1989).

Whole‐exome sequencing (WES), which effectively and efficiently identifies genetic variants in the entire exome, has rarely been used to search for other potential genetic variants associated with VHL disease (Ma et al., 2017). Furthermore, although different variants reportedly correlate with various categories of VHL disease, including type I, type IIA, type IIB, and type IIC, genotype‐phenotype studies performed to date have limited clinical value (Gossage et al., 2015; Linehan, Lerman, & Zbar, 1995). Additionally, researchers have not determined whether other disease‐causing gene variants exist in patients with VHL disease and whether tumor tissues from the same organ share the same driver genes or signaling pathways that promote tumorigenesis. The aforementioned issues are important for clinical diagnosis and treatment. We performed WES and immunohistochemical staining of samples from a family with VHL disease to address these issues.

## 2 METHODS

### 2.1 Ethical compliance

This study was performed in accordance with the principles expressed in the Declaration of Helsinki and was approved by the Institutional Ethics Committee of Peking Union Medical College Hospital, Chinese Academy of Medical Sciences. Written informed consent was obtained from the five participating adults, who agreed to the publication of all their clinical data in this manuscript. For the 3‐year‐old child, informed consent for participation was obtained from the father, who was the proband. The methods were performed in accordance with relevant guidelines and regulations.

### 2.2 Enrolment of family members

**Figure 1:**
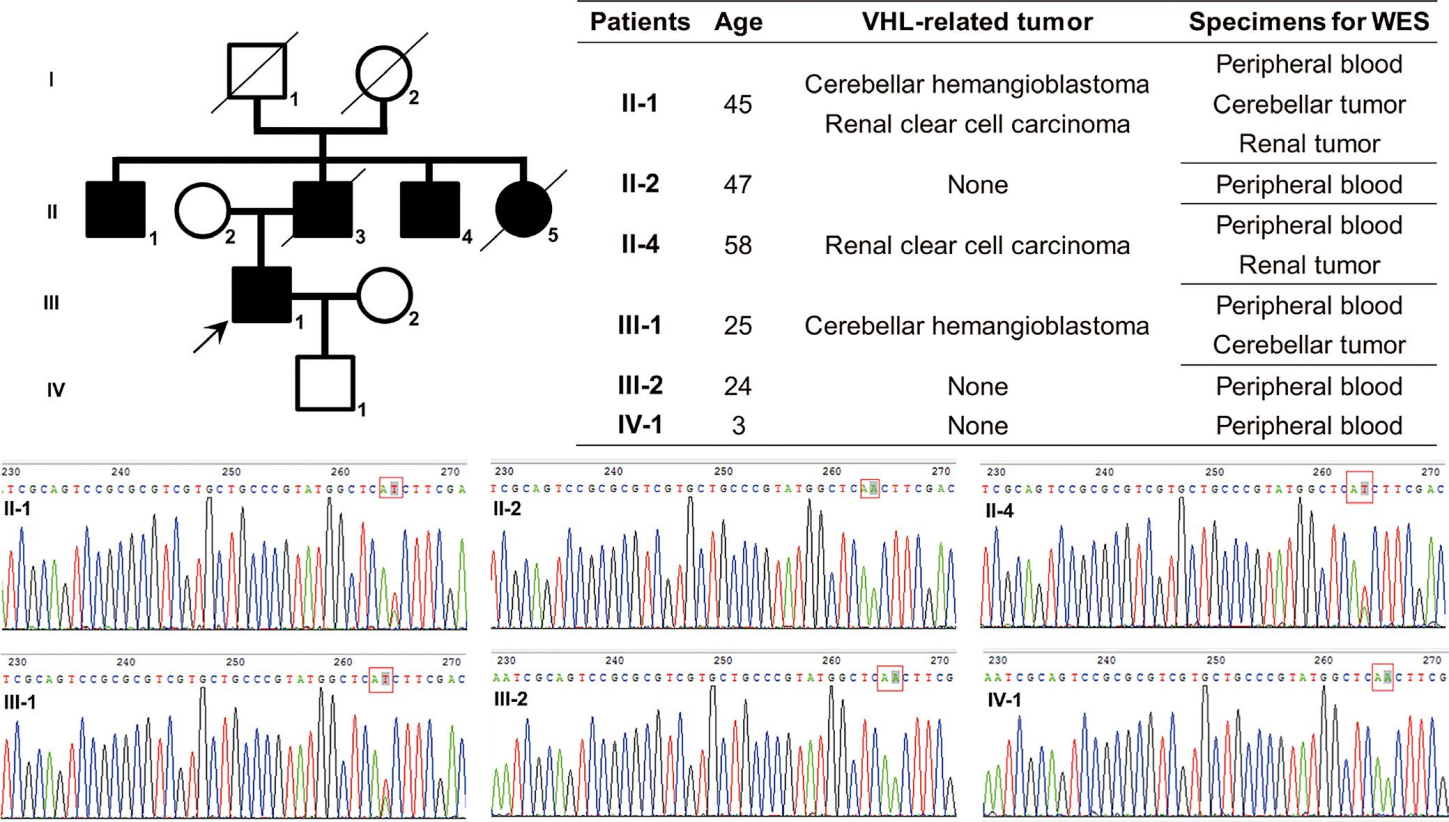
Pedigree chart, VHL‐related tumors, and Sanger sequencing results of *VHL* gene variants using blood samples. The black square and circle represent the patients who were clinically diagnosed with VHL disease. Family members’ ages, VHL‐related tumors and sample sources used for sequencing are listed in the table. The Sanger sequencing results show heterozygous missense variants in the *VHL* gene of Patients III‐1, II‐1, and II‐4

A young male patient (proband) with cerebellar hemangioblastoma (CH) was admitted to the Department of Neurosurgery of Peking Union Medical College Hospital in October 2015. He had a definite family history of VHL disease. His uncle and aunt (deceased) were both diagnosed with CH, and his father (deceased), uncle, and father's elder brother were all diagnosed with RCCC. Considering the existence of both CNS hemangioblastomas and family history, a clinical diagnosis of VHL disease was established. This study included a hematological examination of 6 living family members and an analysis of tumor tissues from 3 patients (Figure 1).

### 2.3 Study design

Sanger sequencing of the VHL gene was performed using peripheral blood samples from 6 family members to initially identify any germline VHL variants. The CH tumor tissue from the proband, the RCCC tumor tissue and CH tumor tissue from his father's elder brother, and the RCCC tumor tissue from his uncle, as well as three peripheral blood samples were subjected to WES to identify somatic gene variants. After detecting novel gene variants, Sanger sequencing was applied for subsequent validation. Immunohistochemical staining for the HIF‐1 and phospho‐S6 ribosomal proteins was performed to assess the expression levels of key proteins.

### 2.4 WES and Sanger sequencing

#### 2.4.1 WES methodology

Total DNA was extracted from peripheral blood, fresh‐frozen tissue samples (from the proband) and formalin‐fixed and paraffin‐embedded (FFPE) tissue samples (from the proband's uncle and father's elder brother) using a UnigeneDx Blood DNA Extraction Kit (UnigeneDx), UnigeneDx Cell/Tissue DNA Extraction Kit (UnigeneDx) and GeneRead DNA FFPE Kit (QIAGEN), respectively. DNA concentrations were determined using the Qubit dsDNA HS assay kit (Life Technologies), and genomic DNA integrity was evaluated using agarose gel electrophoresis.

Exome sequencing libraries were prepared using a KAPA Hyper Prep Kit (KAPA Biosystem, Roche), and enrichment was performed with a SeqCapEZ Exomev3.0 Kit (Nimblegen, Roche) according to the manufacturers’ instructions. Briefly, genomic DNA was sheared to an average size of 200–400 bp using a Covaris M200 sonicator, and approximately 1–1.5 µg of fragmented genomic DNA were used for end‐repair, A‐tailing and adaptor ligation. The samples were barcoded using Illumina‐indexed adaptors.

Size selection was performed before PCR enrichment using AmpureXP beads (Beckman). After library construction, the libraries from the same group of samples (based on DNA quality) were pooled together for capture enrichment. The captured libraries were assessed for enrichment efficiency using an Agilent bioanalyzer and Q‐PCR and sequenced with the Illumina HiSeq platform using 150 bp paired‐end sequencing.

#### 2.4.2 Bioinformatics methodology

An average of 11 Gb of raw data (FASTQ) was generated for each sample using Illumina sequencing. First, the adapter sequences were removed from the raw data, and low‐quality reads with a large number of Ns or a low base quality were discarded. Second, the Burrows‐Wheeler Aligner (Li & Durbin, 2009) was employed to align the sequence reads to the reference genome hg19. SAM‐format files were generated by the Burrows‐Wheeler Aligner. Third, the files in SAM format were further processed to BAM format using Samtools (Li et al., 2009). After these processes, variant calling was performed using GATK (McKenna et al., 2010) and VarScan (Koboldt et al., 2009), and a vcf file was generated. Finally, we used in‐house software to annotate the variants from the vcf file and integrate information from multiple databases. The final variants were fed into the downstream advanced analysis pipeline.

Somatic single‐nucleotide variants (SNVs) are single‐nucleotide variations that occur in any non‐germ cells of the body after conception, possibly initiating tumorigenesis. For somatic variant calling, we also utilized VarScan to identify paired normal (blood) samples and tumor sample‐specific SNVs by simultaneously comparing read counts, base quality, and allele frequencies between blood/normal tissue (B/N) and tumor tissue (T). After SNVs were identified, an annotation was also performed using our in‐house software.

#### 2.4.3 Sanger sequencing

Sanger sequencing was utilized to confirm or refute the nucleotide changes identified using WES, where variants identified using WES but not confirmed by Sanger sequencing were considered false positives. Selected class 3 variants were subjected to Sanger sequencing, and available family members were examined for the presence or absence of the variant. One variant (NM_000551:c.A269T:p.N90I) in the *VHL* gene and two variants (rs62576761 and rs62576762) identified using NGS were confirmed using Sanger sequencing. The pair of primers used to target the variant in the *VHL* gene were Forward Primer CCAGGTCATCTTCTGCAATCG and Reverse Primer TCAGACCGTGCTATCGTCC (NM_000551:c.A269T:p.N90I). The two pairs of primers used for the variants were Forward Primer ATGCCTGGGTCTTGGATAAAC and Reverse Primer GCAACAATCAGGACAGCACAG (rs62576761) and Forward Primer GTGGAAATTTGAGACCAGCAAG and Reverse Primer CAGTGTGTAAGCCAGAAGGG (rs62576762). Variants were confirmed by Sanger sequencing using standard protocols. The sequences of all primers are available upon request.

### 2.5 Immunohistochemical staining

Tumor tissues were stained with hematoxylin and eosin (H&E) to confirm the diagnosis; the tissue sections were reviewed by licensed pathologists to confirm the pathological diagnosis. Markers were immunostained using a previously published protocol (Gao et al., 2016). The anti‐HIF‐1 antibody (Abcam: ab51608) was diluted to 1:200, and the anti‐HIF‐2 antibody (Abcam: ab8365) was diluted to 1:500 with an EDTA antigen retrieval solution (pH 9). The anti‐phospho‐S6 ribosomal protein (Ser235/236) antibody (Cell Signaling Technology: CST4858) was diluted to 1:600 with EDTA antigen retrieval buffer (pH 8).

## 3 RESULTS

### 3.1 Subjects

The pedigree and clinical information are presented in Figure 1. Patient III‐1 was the proband. The proband's father (II‐3), aunt (II‐5), and grandparents (I‐1 and I‐2) were deceased before enrollment.

**Figure 2:**
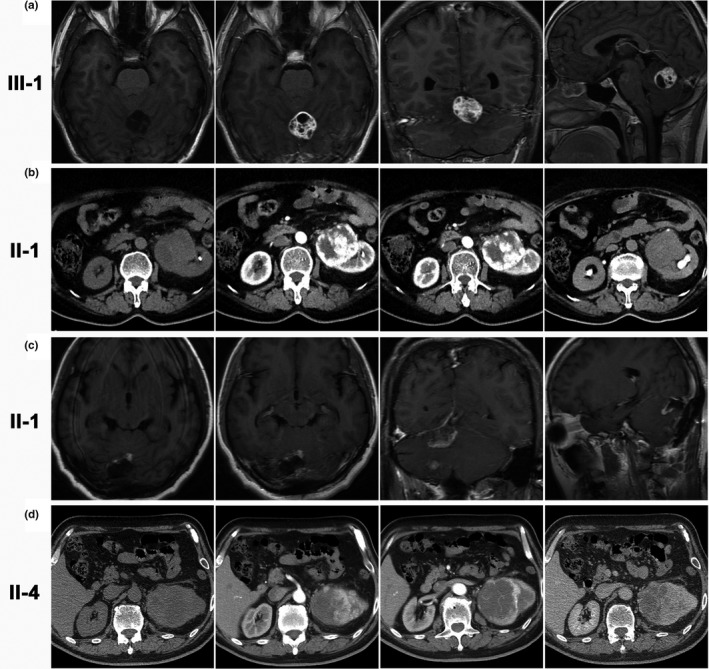
Radiological findings of the four tumors from the three patients. (a) and (c) Images of CH tissues from Patients III‐1 and II‐1 captured using brain MRI. (b) and (d) Abdominal CT images of the RCCC tissues from Patients II‐1 and II‐4. The CH was a mixed mass with both solid and cystic segments. The solid part displayed a isointense/hyperintense signal on T1‐weighted images; the cystic part displayed a hypointense signal. Contrast‐enhanced imaging of the axial, coronal and sagittal planes revealed that the signal of the solid part was markedly enhanced while the cystic part showed a similar hypointense signal to the plain scan. The RCCC originated from the kidney, showing mixed density on plain CT because of hemorrhaging and necrosis inside the fast‐growing tumor. The enhanced CT images revealed a homogenous enhancement in the solid part and no enhancement inside the tumor

The proband (Patient III‐1) was admitted into our ward in October 2015 with a complaint of an occipital headache for 1 month. He was conscious, and a physical examination did not reveal ataxia. Tests for cranial nerve function, limb sensation and motor function were unremarkable. Contrast‐enhanced brain magnetic resonance imaging (MRI) revealed a solid‐cystic mass in the vermis cerebelli and left cerebellar hemisphere (Figure 2a), indicating a radiological diagnosis of CH. Cerebellar tumor resection was performed through a sub‐occipital craniotomy under general anesthesia, and the tumor was completely removed. The tumor, which was 30 mm in diameter, was pink and soft and composed of dilated veins and dense blood capillaries. A postoperative pathological analysis of the tumor indicated a hemangioblastoma. The patient completed outpatient follow‐up in October 2018. The occipital headache had disappeared, brain MRI showed no recurrence, and abdominal computerized tomography (CT) revealed no neoplasms.

Patient II‐1 was admitted to the Department of Urinary Surgery and Neurosurgery in October 2007 and again in December 2015. In the first hospitalization, a serendipitous left kidney mass was found, but no symptoms. Abdominal CT revealed a solid cystic mass on the inside of the left kidney with irregular enhancement (Figure 2b), indicating a radiological diagnosis of RCCC. A laparoscopic radical nephrectomy was performed, and pathology confirmed the diagnosis of clear cell carcinoma. In the second hospitalization, the patient was admitted with complaints of headache and dizziness for 2 months. A brain MRI showed a right CH (Figure 2c). Tumor resection was performed through a posterior approach, and pathology confirmed the diagnosis of hemangioblastoma. In the most recent follow‐up, his symptoms had disappeared, and radiological imaging revealed no recurrence in either the brain or abdomen.

Patient II‐4, the proband's father's elder brother, was admitted to our hospital because of a serendipitous left kidney mass in June 2014. Abdominal CT revealed an obviously enhanced mass on the outside of the left kidney (Figure 2d), indicating a malignant tumor. Kidney tumor resection surgery was performed, and pathology confirmed the diagnosis of clear cell carcinoma. In postoperative follow‐up this year, recurrence was not observed on abdominal CT images.

The proband's son (Patient IV‐1), wife (Patient III‐2), and mother (Patient II‐2) were healthy, and their annual physical examination did not reveal a hemangioblastoma, kidney mass or abdominal cyst. His father (Patient II‐3) died of advanced RCCC in the previous year, his aunt (Patient II‐5) died of a ruptured CH, and his grandparents (Patient I‐1 and I‐2) died from other causes.

We performed Sanger sequencing of the *VHL* gene in six family members. Patients who were clinically diagnosed with VHL disease (Patients III‐1, II‐1, and II‐4) carried heterozygous variants in the *VHL* gene (A to T, c.269 in *VHL* exon 1), whereas no variants were detected in the other three family members (Figure 1).

### 3.2 WES results

We used the human genome build37 (hg19) as the reference genome for this project. In total, 52,850 (III‐1), 52,365 (II‐1), and 52,772 (II‐4) genetic variants were identified in the blood samples using WES. Additionally, 29,431 (II‐1 RCCC), 34,586 (II‐1 CH), 46,787 (II‐4 RCCC), and 52,385 (III‐1 CH) genetic variants were identified in the tumor tissues. According to the bioinformatics analysis, 25,632 (II‐1 RCCC), 31,234 (II‐1 CH), 46,405 (II‐4 RCCC), and 4452 (III‐1 CH) somatic variants were detected in the four tumor tissues.

Moreover, WES revealed some identical gene variants in the same tumor tissues from different patients. Seventeen of the same somatic variants were identified in Patients III‐1 and II‐1 (Supplementary Table S1), and another 10 of the same somatic variants were detected in Patients II‐1 and II‐4 ( Supplementary Table S2). These novel genetic variants may be tissue‐specific variants that can help predict the existence of CH and RCCC based on genetic sequencing. We then searched for novel genetic variants present in all four tumor tissues, revealing two variants in the *SNORD141A* and *SNORD141B* genes in both CH and RCCC tissues (Table 1).

**Table 1:** Identification of the same genetic variants in four tumor samples

Gene	*SNORD141A*	*SNORD141B*
Chromosome	chr9	chr9
Position	135895156	135895217
Reference	A	T
Alteration	T	G
III‐1	319:21:6.18%:0/0|242:28:10.37%:0/1|0.041083132	379:29:7.11%:0/0|299:39:11.54%:0/1|0.024910164
II‐1	294:16:5.16%:0/0|30:8:21.05%:0/1|0.001949366	355:19:5.08%:0/0|35:10:22.22%:0/1|3.26E−04
II‐1	294:16:5.16%:0/0|23:9:28.12%:0/1|1.27E−04	355:19:5.08%:0/0|27:10:27.03%:0/1|6.07E−05
II‐4	344:14:3.91%:0/0|40:19:32.20%:0/1|8.37E**‐**10	393:21:5.07%:0/0|50:22:30.56%:0/1|2.62E**‐**09

### 3.3 Sanger sequencing results

**Figure 3:**
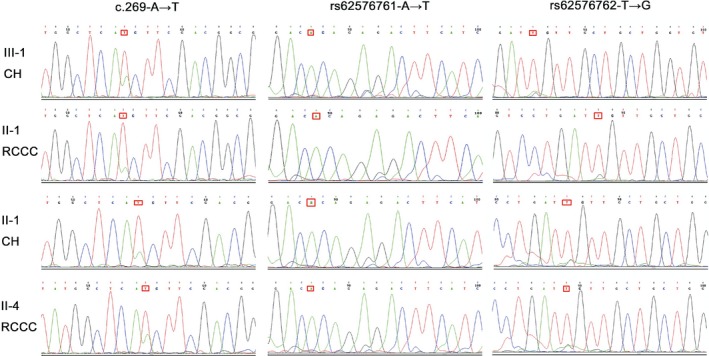
Sanger sequencing results for the *VHL*, *SNORD141A,* and *SNORD141B* variants in tumor samples. *VHL* variants (A to T, c.269 in exon 1) were detected in all samples, whereas no variants in the *SNORD141A* and *SNORD141B* genes were identified

We then performed Sanger sequencing of *SNORD141A* (rs62576761), *SNORD141B* (rs62576761) and *VHL* (c.269) to confirm these genetic variants identified using WES. Although the Sanger sequencing results revealed no variants in *SNORD141A* or *SNORD141B* (Figure 3), heterozygous variants in the *VHL* gene were confirmed in all four of the tumor tissues.

### 3.4 Immunohistochemical staining and pathogenicity analysis

**Figure 4:**
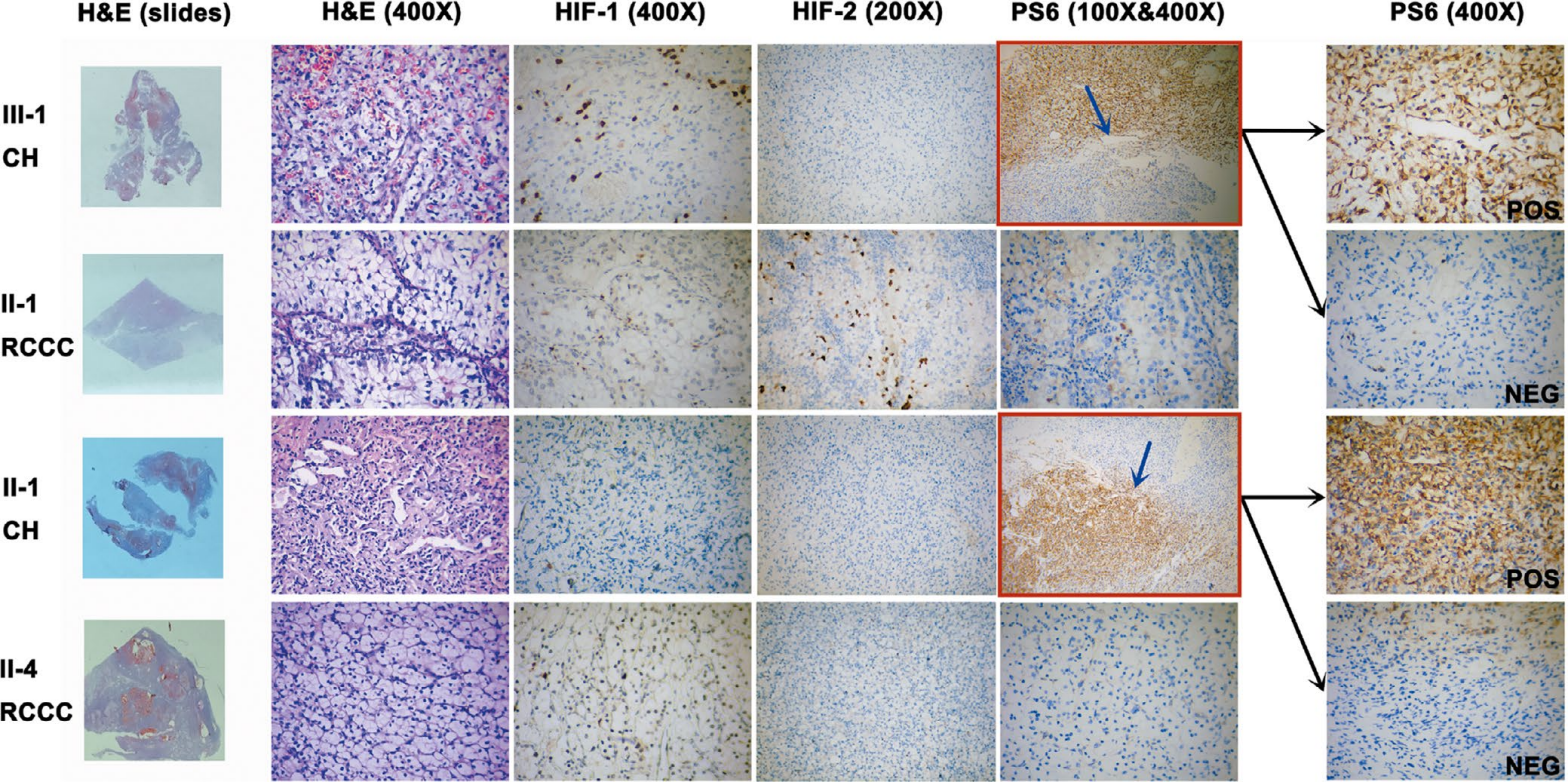
H&E staining and immunohistochemical staining for HIF‐1, HIF‐2, and PS6. H&E staining showed large stromal cells admixed with the thin‐walled capillary network in CH and tumor cells with an abundant, clear cytoplasm in RCCC. HIF‐1α and HIF‐2α staining was negative in the tumor cell compartment; positively stained cells were mainly interstitial and capillary vessel cells. RCCC tissues were negative for PS6 staining, but CH was lobular positive. A clear demarcation divided the CH tissue into two parts in both patients: a PS6‐positive tissue and PS6‐negative tissue

The *VHL* gene variants were confirmed in both the tumor tissues and blood samples from all patients who were clinically diagnosed with VHL disease. Immunohistochemical staining was performed for the HIF‐1α (Abcam: ab51608), HIF‐2α (Abcam: ab8365) and phospho‐S6 ribosomal proteins (Ser235/236) (Cell Signaling: CST4858). Vascular‐interstitial cells, but not stromal cells, displayed HIF‐1α‐ and HIF‐2α‐positive staining. The RCCC tissue was negative for pS6 protein staining, whereas staining was “lobular” positive in the CH tissues, with a clear demarcation (Figure 4).

## 4 DISCUSSION

The family analyzed in the present study exhibits typical type 1 familial VHL disease, without evidence of pheochromocytomas (Chen et al., 1995). Our experiments using WES and Sanger sequencing did not detect other significant somatic SNVs in VHL‐related tumors, except for the *VHL* c.269 A → T variant.

The pVHL tumor suppressor protein regulates the levels of HIF proteins (Maxwell et al., 1999) in response to hypoxic conditions. pVHL directly binds to HIF1α or HIF2α, leading to the hydroxylation and ubiquitination of the HIFα protein (Yu, White, Zhao, & Lee, 2001). However, when pVHL function is lost, HIFα accumulates and binds to HIF1β to exert its biological functions. In the present study, HIF‐1α and HIF‐2α were examined as representative protein targets of pVHL, and positive staining was observed for both proteins in vascular‐interstitial cells, but not in stromal cells. This phenomenon might be partially explained by the incomplete LOH events that occurred in the tumor tissue.

We also studied the mTOR pathway in tumor tissues. The regulatory mechanism of HIF‐1 and mTOR has been documented, with HIF‐1 activation followed by downregulation of mTOR (Brugarolas et al., 2004). According to other studies, HIF2α activates mTOR signaling via SLC7A5 (Elorza et al., 2012). Interestingly, lobular‐positive samples were identified in cerebellar hemangioblastoma tissues, but samples of renal clear cell cancer tissues were negative. To the best of our knowledge, mTOR activation in cerebellar hemangioblastomas is rare. Based on our results, mTOR signaling might play an important role in the development of cerebellar hemangioblastoma in patients with VHL disease and may be a target for future research on VHL‐related cerebellum hemangioblastomas.

This study has some limitations. First, as CNVs of VHL‐related tumors were not analyzed in this study, we are not able to exclude the impact of CNVs on VHL tumorigenesis. Second, based on the small number of cases, some potential somatic and pathological factors might not have been identified.

## 5 CONCLUSIONS

In this pedigree, VHL‐related tumors exhibited no other genetic SNV in WES, although CNV involvement in VHL tumorigenesis cannot be excluded. The mTOR pathway is a potential target for VHL‐related hemangioblastomas.

## CONFLICT OF INTEREST STATEMENT

The authors have no competing interests to declare.

## ETHICAL APPROVAL AND INFORMED CONSENT

This study was performed in accordance with the principles expressed in the Declaration of Helsinki and was approved by the Institutional Ethics Committee of Peking Union Medical College Hospital, Chinese Academy of Medical Sciences. Written informed consent was obtained from the 5 participating adults, and they agreed to the publication of all their clinical data in this manuscript. Informed consent for participation by the 3‐year‐old child was obtained from his father, the proband. The methods were performed in accordance with relevant guidelines and regulations.

## AUTHORS’ CONTRIBUTIONS

X.G., L.G., and X.H. performed the genetic studies and literature review. X.G., X.H., D.G., W.D., and X.W. performed the variant analysis and created the figures for the manuscript. X.G., L.G., X.H., and Z.X. participated in writing the manuscript. B.X. designed and supervised the study and revised the manuscript. All authors read and approved the final manuscript.

6

## Data Availability

The datasets used and/or analyzed during the current study are available from the corresponding author upon reasonable request.
