# Supplementary material for: Whole‐exome sequencing and immunohistochemistry findings in von Hippel–Lindau disease
Source: Mol Genet Genomic Med. 2019 Jul 17;7(9):e880. doi: 10.1002/mgg3.880 (PMC6732316; doi:10.1002/mgg3.880)
Supplement: Supplementary file 1 [file MGG3-7-e880-s001.docx]

**Supplementary Table 1.** Identification of the same genetic mutations in the CH tissues from Patients III-1 and II-1.

| **Chr** | **Position** | **Ref** | **Alt** | **Gene** | **CH from Patient III-1** | **CH from Patient II-1** |
| --- | --- | --- | --- | --- | --- | --- |
| chr15 | 21324357 | G | A | *LINC01193* | 168:15:8.20%:0/0\|163:28:14.66%:0/1\|0.035556946 | 144:11:7.10%:0/0\|21:18:46.15%:0/1\|5.30E-08 |
| chr15 | 21324402 | A | G | *LOC646214* | 138:10:6.76%:0/0\|131:20:13.25%:0/1\|0.046253013 | 108:7:6.09%:0/0\|14:11:44%:0/1\|9.90E-06 |
| chr15 | 43856300 | C | T | *PPIP5K1* | 167:10:5.65%:0/0\|114:17:12.98%:0/1\|0.020953492 | 124:8:6.06%:0/0\|25:6:19.35%:0/1\|0.028562825 |
| chr15 | 43856308 | C | T | *PPIP5K1* | 166:10:5.68%:0/0\|122:17:12.23%:0/1\|0.031846135 | 129:7:5.15%:0/0\|24:6:20%:0/1\|0.014565809 |
| chr15 | 43856323 | C | T | *PPIP5K1* | 166:8:4.60%:0/0\|127:16:11.19%:0/1\|0.023075646 | 140:6:4.11%:0/0\|25:5:16.67%:0/1\|0.022688882 |
| chr15 | 43856336 | C | G | *PPIP5K1* | 168:8:4.55%:0/0\|127:15:10.56%:0/1\|0.032917131 | 140:6:4.11%:0/0\|27:5:15.62%:0/1\|0.028641681 |
| chr16 | 64727 | C | T | *DDX11L10* | 219:13:5.60%:0/0\|275:36:11.58%:0/1\|0.010962948 | 226:18:7.38%:0/0\|21:6:22.22%:0/1\|0.021207204 |
| chr16 | 89017636 | G | C | *LOC100129697* | 244:14:5.43%:0/0\|167:20:10.70%:0/1\|0.030473273 | 218:18:7.63%:0/0\|16:6:27.27%:0/1\|0.009313684 |
| chr20 | 29628328 | C | G | *FRG1BP, FRG1DP* | 769:68:8.12%:0/0\|656:86:11.59%:0/1\|0.012858517 | 582:47:7.47%:0/0\|160:29:15.34%:0/1\|0.001397462 |
| chr20 | 43851524 | T | A | *SEMG2* | 107:2:1.83%:0/0\|88:10:10.20%:0/1\|0.010224288 | 93:2:2.11%:0/0\|19:4:17.39%:0/1\|0.012999483 |
| chr22 | 16635626 | G | C | *OR11H1* | 437:33:7.02%:0/0\|347:43:11.03%:0/1\|0.026470402 | 266:28:9.52%:0/0\|31:17:35.42%:0/1\|1.34E-05 |
| chr7 | 102898150 | G | A | *DPY19L2P2* | 135:5:3.57%:0/0\|122:15:10.95%:0/1\|0.015151317 | 133:13:8.90%:0/0\|40:12:23.08%:0/1\|0.010524549 |
| chr7 | 822072 | C | G | *DNAAF5* | 134:10:6.94%:0/0\|66:12:15.38%:0/1\|0.040270557 | 99:10:9.17%:0/0\|49:15:23.44%:0/1\|0.010238678 |
| chr9 | 135895156 | A | T | *SNORD141A, SNORD141B* | 319:21:6.18%:0/0\|242:28:10.37%:0/1\|0.041083132 | 294:16:5.16%:0/0\|30:8:21.05%:0/1\|0.001949366 |
| chr9 | 135895217 | T | G | *SNORD141A, SNORD141B* | 379:29:7.11%:0/0\|299:39:11.54%:0/1\|0.024910164 | 355:19:5.08%:0/0\|35:10:22.22%:0/1\|3.26E-04 |
| chr9 | 45002277 | C | T | *LOC102723709* | 465:39:7.74%:0/0\|452:56:11.02%:0/1\|0.04585043 | 511:33:6.07%:0/0\|46:15:24.59%:0/1\|1.75E-05 |
| chr9 | 69425782 | G | A | *ANKRD20A4* | 543:52:8.74%:0/0\|518:71:12.05%:0/1\|0.037891161 | 647:60:8.49%:0/0\|114:28:19.72%:0/1\|1.62E-04 |
