# Supplementary material for: Whole‐exome sequencing and immunohistochemistry findings in von Hippel–Lindau disease
Source: Mol Genet Genomic Med. 2019 Jul 17;7(9):e880. doi: 10.1002/mgg3.880 (PMC6732316; doi:10.1002/mgg3.880)
Supplement: Supplementary file 2 [file MGG3-7-e880-s002.docx]

**Supplementary Table 2.** The same genetic mutations in the RCC tissues from Patients II-1 and II-4.

| **Chr** | **Position** | **Ref** | **Alt** | **Gene** | **RCC from Patient II-1** | **RCC from Patient II-4** |
| --- | --- | --- | --- | --- | --- | --- |
| chr12 | 8326972 | G | A | *ZNF705A* | 421:35:7.68%:0/0\|17:12:41.38%:0/1\|2.60E-06 | 561:30:5.08%:0/0\|88:12:12%:0/1\|0.01107023 |
| chr17 | 29587482 | G | A | *NF1* | 120:0:0%:0/0\|13:2:13.33%:0/1\|0.011608624 | 157:0:0%:0/0\|16:2:11.11%:0/1\|0.010049261 |
| chr9 | 123261 | C | T | *CBWD1* | 616:22:3.45%:0/0\|52:6:10.34%:0/1\|0.022846311 | 941:19:1.98%:0/0\|69:8:10.39%:0/1\|4.50E-04 |
| chr10 | 43318589 | A | C | *BMS1* | 469:22:4.48%:0/0\|121:14:10.37%:0/1\|0.011423408 | 598:16:2.61%:0/0\|149:18:10.78%:0/1\|3.39E-05 |
| chr12 | 52886641 | C | T | *KRT6A* | 264:9:3.30%:0/0\|11:5:31.25%:0/1\|4.00E-04 | 347:10:2.80%:0/0\|73:9:10.98%:0/1\|0.003354172 |
| chr12 | 52886711 | C | T | *KRT6A* | 219:19:7.98%:0/0\|6:4:40%:0/1\|0.008213794 | 307:13:4.06%:0/0\|55:8:12.70%:0/1\|0.011983119 |
| chr14 | 19553612 | C | T | *POTEG* | 582:49:7.77%:0/0\|12:4:25%:0/1\|0.03487259 | 391:17:4.17%:0/0\|40:9:18.37%:0/1\|6.89E-04 |
| chr11 | 71544246 | A | G | *DEFB108B* | 102:2:1.92%:0/0\|18:6:25%:0/1\|5.30E-04 | 165:6:3.51%:0/0\|47:8:14.55%:0/1\|0.006830133 |
| chr9 | 135895156 | A | T | *SNORD141A* | 294:16:5.16%:0/0\|23:9:28.12%:0/1\|1.27E-04 | 344:14:3.91%:0/0\|40:19:32.20%:0/1\|8.37E-10 |
| chr9 | 135895217 | T | G | *SNORD141B* | 355:19:5.08%:0/0\|27:10:27.03%:0/1\|6.07E-05 | 393:21:5.07%:0/0\|50:22:30.56%:0/1\|2.62E-09 |
